# Effectiveness and Safety of JAK Inhibitors in Autoinflammatory Diseases: A Systematic Review

**DOI:** 10.3389/fmed.2022.930071

**Published:** 2022-06-27

**Authors:** Zhivana Boyadzhieva, Nikolas Ruffer, Gerd Burmester, Anne Pankow, Martin Krusche

**Affiliations:** ^1^Department of Rheumatology and Clinical Immunology, Charité—Universitätsmedizin Berlin, Berlin, Germany; ^2^Division of Rheumatology and Systemic Inflammatory Diseases, University Hospital Hamburg-Eppendorf (UKE), Hamburg, Germany

**Keywords:** autoinflammation, interferonopathy, monogenic autoinflammatory disease, Janus Kinase inhibition, *innate immunity*

## Abstract

**Introduction:**

Autoinflammatory diseases (AID) are rare diseases presenting with episodes of sterile inflammation. These involve multiple organs and can cause both acute organ damage and serious long-term effects, like amyloidosis. Disease-specific anti-inflammatory therapeutic strategies are established for some AID. However, their clinical course frequently includes relapsing, uncontrolled conditions. Therefore, new therapeutic approaches are needed. Janus Kinase inhibitors (JAKi) block key cytokines of AID pathogenesis and can be a potential option.

**Methods:**

A systematic review of the literature in accordance with the PRISMA guidelines was conducted. Three databases (MEDLINE, Embase and Cochrane Central Register of Controlled Trials) were searched for publications regarding the use of JAKi for AID. Data from the included publications was extracted and a narrative synthesis was performed. Criteria for defining treatment response were defined and applied.

**Results:**

We report data from 38 publications with a total of 101 patients describing the effects of JAKi in AID. Data on Type I Interferonopathies, Adult-Onset Still's Disease (AOSD), Systemic Juvenile Idiopathic Arthritis (sJIA), Familial Mediterranean Fever (FMF), and Behçet's Syndrome (BS) was identified. From a total of 52 patients with type I interferonopathies, in seven patients (7/52, 13.5%) a complete response was achieved, most (35/52, 67.3%) showed a partial response and a minority (10/52, 19.2%) showed no treatment response. For AOSD, a complete or a partial response was achieved by eleven (11/26, 42.3%) patients each. Two sJIA patients achieved complete response (2/4, 50%) and in two cases (2/4, 50%) a partial response was reported. Half of FMF patients showed a complete response and the other half had a partial one (3/6, 50.0%). Amongst BS patients most achieved a partial response (8/13, 61.5%). Five patients showed no response to therapy (5/13, 38.5%). Overall, the most frequent AEs were upper respiratory tract infections (17), pneumonia (10), BK virus viremia (10) and viruria (4), herpes zoster infection (5), viral gastroenteritis (2) and other infections (4).

**Conclusion:**

The results from this systematic review show that JAKi can be beneficial in certain AID. The risk of AEs, especially viral infections, should be considered. To accurately assess the risk benefit ratio of JAKi for AID, clinical trials should be conducted.

## Introduction

Autoinflammatory diseases (AID) are characterized by seemingly unprovoked inflammatory attacks in absence of pathogenic autoantibodies or antigen-specific T-cells. Defined as *mono- and polygenic disorders of innate immunity*, AID comprise a broad spectrum of rare diseases which may present with episodes of fever and sterile inflammation potentially causing severe morbidity and mortality. Due to advances in gene sequencing technology and the development of diagnostic criteria, new syndromes continue emerging (1, 2).

Depending on the dominating cytokine pattern, AID can be grouped in IL-1 (inflammasomopathies) (3), NFκB (relopathies) (4) or type I interferon (IFN)-driven diseases (interferonopathies) (5). However, in multiple syndromes such as Adult-Onset Still's Disease [AOSD; IL-1, IL-6, IL-18 (6, 7)], Behçet's syndrome [BS; IL-1, IL-6 (8), IFNγ (9)] or Familial Mediterranean Fever [FMF; IL-1 (10) and IL6 (11)] more than one cytokine plays a key role in pathogenesis. Due to the broad disturbance of cytokine signaling, AID can affect various organs and are thus associated with a high disease burden and severe physical, but also socioeconomic limitations (12). Furthermore, AID patients with persistent inflammation have a high risk of developing AA amyloidosis (13, 14).

Current management of AID includes targeted inhibition of specific cytokine signaling. For example, targeted IL-1 inhibition has been shown to be effective for some conditions such as cryopyrin-associated periodic syndromes (CAPS) (15) and AOSD (16). Unfortunately, some AID patients do not respond to targeted inhibition of specific cytokines and other treatment options are needed (17).

Janus Kinase inhibitors (JAK inhibitors, JAKi) interfere with signal transduction of the Janus Kinase-Signal transducer and activator of transcription (JAK-STAT) pathway causing effective suppression of downstream cytokine signaling. JAK-STAT signaling can be triggered by two types of cytokine receptors: type I receptors bind mainly cytokines (IL-2,−6,−9,−12,−15), hormones (growth hormone, GH) and colony stimulating factors, while type 2 receptors are activated mostly by interferon and IL-10 (18). They act as competitive antagonists at activation sites for Janus kinases and as such interrupt downstream signals along the JAK-STAT pathway, effectively leading to suppression of cytokine production. The JAK-STAT pathway includes several kinases and JAKi can be grouped by their kinase-specific effects: tofacitinib—JAK1, JAK2 and JAK3, baricitinib and ruxolitinib- selective inhibition of JAK1 and JAK2, upadacitinib and filgotinib—selective for JAK1. While JAKi are considered as “targeted therapies,” there is almost no other substance class that exerts an effect on such a large number of cytokines. The resulting immunomodulatory effects can be clinically illustrated by the fact that the drugs have already been approved for a number of rheumatic diseases such as rheumatoid arthritis (RA), psoriatic arthritis (19, 20), polyarticular juvenile arthritis (tofacitinib) (21) and ankylosing spondylitis (upadacitinib, tofacitinib) (22, 23).

First reports from an expanded access program study on the beneficial effects of JAKi in type I interferonopathies (24) have also been published. Due to their broad blockade of proinflammatory pathways, JAKi may ameliorate autoinflammatory processes and thus lead to clinical remission in otherwise refractory AID cases. The aim of this systematic literature review is to identify and analyze the available evidence on JAKi for the treatment of autoinflammatory diseases.

## Methods

The Preferred Reporting Items for Systematic Reviews and Meta-Analyses guidelines were followed for preparing the manuscript (25).

### Protocol and Registration

A study protocol was registered at PROSPERO (CRD42021270369) prior to the systematic search (26).

### Data Sources and Searches

The following databases were systematically searched for publications investigating the role of JAKi in AID treatment: MEDLINE *via* PubMed, EMBASE *via* Ovid, Cochrane Central Register of Controlled Trials (via Cochrane Library). The search was conducted on 30 June 2021 and updated on 16 October 2021. The results were supplemented by a backwards search of relevant publications (reference screening).

The search strings were built based on two components using the Boolean operator *and* (AID *and* JAKi). Within those components, multiple terms were linked by *or*. For each syndrome, the full and the abbreviated terms were used including at least one synonym for each condition. For MEDLINE both Medical Subject Headings (MeSH) terms and free-text words were used. All keywords were used to search within titles and abstracts of publications.

Details of the complete search strategy for all searched databases can be found in the Supplementary Materials.

### Study Selection

Criteria for inclusion were developed using the Patient, Intervention, Comparator, Outcome (PICO) scheme (27). Of interest were following diseases/syndromes:

- Adult-Onset Still's disease (AOSD)
- Systemic Juvenile Idiopathic Arthritis (sJIA)
- Familial Mediterranean Fever (FMF)
- Cryopyrin-associated Periodic Syndromes (CAPS)
- TNF-Receptor Associated Periodic Syndrome (TRAPS)
- Mevalonate Kinase Deficiency (MKD)
- Pyogenic Arthritis, Pyoderma Gangrenosum and Acne (PAPA) Syndrome
- Periodic Fever, Aphthous Stomatitis, Pharyngitis and Adenitis (PFAPA) Syndrome
- Genetic Interferonopathies: Aicardi Goutières Syndrome (AGS), Chronic atypical neutrophilic dermatosis with lipodystrophy and elevated temperature (CANDLE) Syndrome, STING associated vasculitis with onset in infancy (SAVI) Syndrome
- Behçet's Syndrome

Defined as *Intervention* was the usage of JAKi (tofacitinib, upadacitinib, baricitinib, filgotinib or ruxolitinib). As *Comparator* we accepted any other treatment. For *Outcome* we analyzed treatment response (see below) and safety (considered were reports on any adverse events).

No restrictions were applied concerning publication date, age, and number of recruited patients. Only studies published in English were included. Considered for inclusion were both retrospective (e.g., case reports, case-series, case-control studies) and prospective studies (e.g., randomized controlled trials, non-randomized controlled trials, prospective observational studies).

Assessment for eligibility was performed by two independent reviewers (AP and ZB), following inclusion and exclusion criteria (Table 1). First, only title and abstract were screened. Suitable publications were then assessed in full text. Where there were discrepancies in the evaluation of the eligibility of a publication by the two reviewers, a third reviewer acted as an arbiter (MK).

**Table 1:** Inclusion and exclusion criteria.

**Inclusion criteria**	**Exclusion criteria**
a) *Patient population*: Patients with AID (AOSD, sJIA, FMF, BS, CAPS, TRAPS, PAPA, PFAPA, Type I Interferonopathies) b) *Intervention*: tofacitinib, baricitinib, upadacitinib, filgotinib, ruxolitinib, other JAKi c) *Comparators:* any other treatment d) *Outcomes*: effectiveness, safety e)*Study design*: retrospective (e.g., case reports, case-series, case-control studies, cohort studies); prospective studies (e.g., randomized controlled trials, non-randomized controlled trials, prospective observational studies) f) *Language*: English	a) *Patient population*: animal/ *in-vitro* study, AID other than specified b) *Intervention*: other than specified c) *Outcomes*: no/insufficient clinical results d) *Publication type:* review articles e) *Language*: other

### Data Collection Process and Data Items

Data extraction and management was performed with Microsoft Excel 2016. A standardized data extraction sheet was designed and used for extraction of study characteristics and outcome data, which was carried out by one of the reviewers (ZB). Data was extracted from each publication on: (1) study characteristics; (2) patient characteristics at baseline; (3) patient characteristics after intervention.

### Summary Measures, Synthesis

Due to the lack of randomized controlled trials and the heterogeneity of data, a narrative synthesis was carried out. Results were reported based on the Synthesis Without Meta-analysis (SWiM) guideline (28).

Here, the treatment response of each patient was classified as *complete, partial* or *none* based on the available data on clinical symptoms and laboratory parameters prior/post intervention. A *complete* response was defined as resolution of all clinical symptoms and normalization of inflammatory parameters (Erythrocyte Sedimentation Rate, ESR, and/or C-Reactive Protein, CRP); as *partial* when either clinical symptoms resolved or laboratory markers normalized, and as *none* when both remained unchanged or worsened.

## Results

The first database search identified 582 records of which 70 were removed (duplicate records). The 512 records were screened. Reference screening of included publications additionally identified 4 suitable publications. The updated search (June to October 2021) identified further 80 publications, of which 75 were screened.

**Figure 1:**
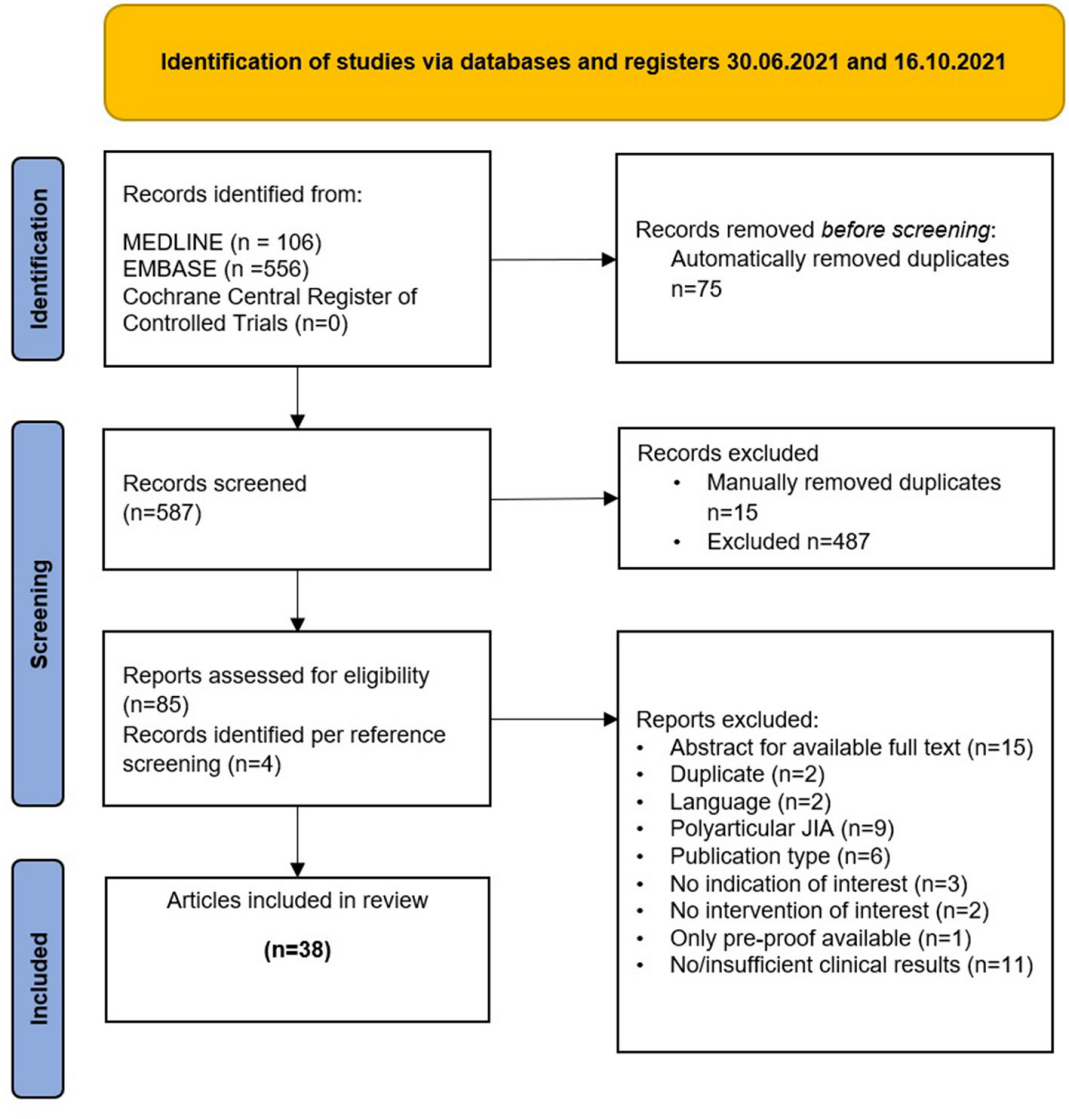
Identification of studies *via* databases and registers 30.06.2021 and 16.10.2021.

Overall, 38 original publications were included for data extraction and analysis. A total of 101 AID patients treated with a JAKi could be identified. Figure 1 provides details on the selection process of included studies.

### Evidence on Effectiveness and Safety for Chronic Atypical Neutrophilic Dermatosis With Lipodystrophy and Elevated Temperature (CANDLE) Syndrome

The database search identified four case reports (full text *n* = 3, conference abstracts *n* = 1) (29–32) and two articles reporting results of the same compassionate use study (24, 33). A total of fourteen patients were treated with a JAKi. Median age of JAKi initiation was 8.5 years (1.5–17 years, reported for 4 patients). Eleven patients received baricitinib (11/13, 84.6%), and three received tofacitinib (3/14, 21.4%). Mean treatment duration was 92.4 months (reported for 13 patients). All but one patient received glucocorticoids (GC) in addition to a JAKi. Data on baseline characteristics, treatment and response are shown in Supplementary Table 1.

Six of the patients (6/14, 42.9%) had a complete response to therapy, half (7/14, 50.0%) showed a partial response and one (1/14, 7.1%) did not respond at all. GC dosage at the end of follow up was reported for eleven patients (11/13, 84.6%), of whom seven (7/11, 63.6%) successfully discontinued GC. In four patients (4/11, 36.4%) GC dose reduction was possible.

Data on adverse events (AEs) was available for thirteen patients (13/14, 92.9%). One patient experienced transient muscle pain. One other developed gamma-GT elevation with dyslipidemia. The latter was managed with atorvastatin. Both cases did not require therapy discontinuation. The most common AEs were infections: BK virus viremia (6/13, 46.2%), herpes zoster (2/13, 15.4%), upper respiratory tract infections (UTI) (10/13, 76.9%) and pneumonia (4/13, 30.8%) none of which required treatment discontinuation. Of all AEs hospitalization was required in 3 cases (3/25, 12%): for BK viremia, herpes zoster and pneumonia.

One patient discontinued therapy after 67.5 months because of acute kidney injury following a series of infections (pneumocystis jirovecii pneumonia, clostridium difficile, influenza, and rotavirus). Details on AEs are summarized in Table 2.

**Table 2:** Overview of adverse events.

**Disease**	**CANDLE**	**SAVI**	**AGS**	**Other type I interferonopathies**	**AOSD**	**sJIA**	**FMF**	**BS**	**Total**
Number of patients treated	14	28	3	7	26	4	6	13	101
Adverse events—n*	26	17	1	9	4	0	0	2	59
JAKi dose reduction	–	2	–	–	–	–	–	–	2
JAKi discontinuation	1	2	–	–	1	–	–	2	6
requiring hospitalization	3	4	–	2	–	–	–	–	9
Deaths	–	4**	–	–	1***	–	–	–	5
Types of adverse events
Pneumonia	4	3	–	–	3	–	–	–	10
Dose reduction	–	1	–	–	–	–	–	–	1
Discontinuation	–	–	–	–	–	–		–	
UTI	10	5	–	2	–	–	–	–	17
Dose reduction	–	–	–	–	–	–	–	–	–
Discontinuation	–	–	–	–	–	–	–	–	–
BK viremia	6	3	–	1	–	–	–	–	10
Dose reduction	–	1	–	–	–	–	–	–	1
Discontinuation	–		–	–	–	–	–	–	–
BK viruria	–	1	–	3	–	–	–	–	4
Dose reduction	–	–	–	–	–	–	–	–	–
Discontinuation	–	–	–	–	–	–	–	–	–
Herpes zoster	2	–	–	1	–	–	–	2	5
Dose reduction	–	–	–	1	–	–		–	1
Discontinuation	–	–	–	–	–	–	–	2	2
Viral gastroenteritis	**–**	2	**–**	**–**	**–**	**–**	**–**	**–**	2
Dose reduction	–	–	–	–	–	–	–	–	–
Discontinuation	–	1	–	–	–	–	–	–	1
Other infections	1^a^	2^b^	–	1^c^	–	–	–	–	4
Dose reduction	–	–	–	–	–	–	–	–	–
Discontinuation	–	–	–	–	–	–	–	–	–
Dyslipidemia	1	–	1	–	–	–	–	–	2
Dose reduction	–	–	–	–	–	–	–	–	–
Discontinuation	–	–	–	–	–	–	–	–	–
Other AEs	2	1	–	1	1^g^	–	–	–	5
Dose reduction	–		–	–	–	–	–	–	–
Discontinuation	1^d^	1^e^	–	1^f^	1	–	–	–	4

^*^Information on adverse events was available for 13 CANDLE patients, 20 SAVI patients, 1 AGS patient, 5 patients with other interferonopathies, 24 AOSD patients, 3 sJIA patients, 4 FMF patients and all 13 Behçet's Syndrome patients.

^**^One due to ILD and heart failure; one after humoral rejection after lung transplant due to ILD; one due to acute respiratory failure; one due to ILD.

^***^One of the patients with bacterial pneumonia, after a 217-day long hospital stay.

^a^*multiple: pneumocystis jirovecii pneumonia, clostridium difficile, influenza, and rotavirus; ^b^osteomyelitis; cutaneous infection with staphylococcus aureus; ^c^multiple: clostridium difficile, pyelonephritis, urosepsis; ^d^acute kidney injury; ^e^papillary edema; ^f^osteonecrosis; ^g^menometrorrhagia*.

### Evidence on Effectiveness and Safety for STING Associated Vasculopathy With Onset in Infancy (SAVI) Syndrome

Six case reports (34–39) and seven case series (40–46) reporting on SAVI could be identified (full text *n* = 9, conference abstracts *n* = 1, letters *n* = 3). Additional two articles (24, 33) reported on the same study population (patients with SAVI, CANDLE, other interferonopathies).

Data was extracted and analyzed for a total of twenty-eight patients. Median age of JAKi initiation was 7.5 years (1 month-37 years, reported for 24 patients). Eighteen patients (18/28, 64.3%) received ruxolitinib, seven patients (7/28, 25%) received baricitinib, and three (3/28, 10.7%) received tofacitinib. Mean treatment duration was 23.7 months (2.5–80.1 months, reported for 27 patients). For five patients (5/28, 17.9%) data on supportive treatment was not available. A minority of patients (6/23, 26.1% of reported cases) received JAKi monotherapy. Most were on concomitant GC (16/23, 69.6% of reported cases) of whom six (5/16, 31.3% of reported cases) also received additional immunosuppression (e.g., hydroxychloroquine, IVIG, etanercept). One patient received only IVIG in combination with JAKi (1/23, 4.3%). A summary of baseline characteristics, treatment and response is shown in Supplementary Table 1. A quarter of the patients (7/28, 25%) showed no clinical and laboratory response. While most patients experienced improvement either in clinical symptoms, or in laboratory parameters of inflammation, no patient achieved complete remission. GC dosage at last follow-up was reported for twelve patients (12/16, 75%). For eight patients (8/12, 66.7% of reported cases) complete tapering was possible and in three cases (3/12, 25%) GC dose reduction was tolerated.

Data on AEs was available for twenty patients (20/28, 71.4%) and were mostly infectious: UTI (4), pneumonia (3), osteomyelitis (1), cutaneous infection (1), BK viremia (2), BK viruria (1) and gastroenteritis (1) all without the need for therapy discontinuation or reports of dose reduction. JAKi dose reduction was required in two cases: after recurring respiratory infections and in one case of BK viremia. Treatment discontinuation occurred in two cases (severe rotavirus enteritis, papillary edema). In both cases, JAKi therapy was later reinstated and well-tolerated. Four patients (4/28, 14.3%) died during JAKi treatment. Of all AEs, one (enteritis) occurred while the patient was hospitalized. Hospitalization was otherwise required in four cases (4/17, 23.5%): for gastroenteritis, two cases of pneumonia and recurring respiratory infections. Details on AEs are summarized in Table 2.

### Evidence on Effectiveness and Safety for Aicardi Goutières Syndrome (AGS)

The systematic searches identified three case reports on AGS (full text *n* = 2, letters *n* = 1) (47–49). One additional letter reports preliminary results of an open-label single center study involving 35 patients with AGS (50). The publication is discussed in the Discussion section since no individual patient data was available.

Here, we report the available data on two pediatric patients and one adult. The median age of JAKi initiation was 11 years (1.5–22 years). The patients were treated for a mean duration of 28.3 months (18–43 months). A summary of baseline characteristics, treatment and response is shown in Supplementary Table 1. All patients (3/3, 100%) showed a partial response to therapy.

Data on AEs was available for only one patient (1/3, 33.3%) (48) who developed creatine kinase fluctuations, hypercholesterinemia, and hypertriglyceridemia, which were transient and controlled by dietary management without the need for JAKi dose reduction or hospitalization. No other AEs were reported. Details on AEs are summarized in Table 2.

### Evidence on Effectiveness and Safety for Other Type I Interferonopathies

Three case reports (51–53) (conference abstracts *n* = 1, full text *n* = 2) and two articles reporting results of the same compassionate use study (24, 33) regarding type I interferonopathies were identified.

A total of seven patients were treated. One patient was diagnosed with DNase II deficiency. For the rest either only “other type I interferonopathy” was reported as diagnosis or a novel mutation was described (Table 1). Median age of JAKi initiation was 4 years (1 month −17 years, reported for 3 patients). Five patients (5/7, 71.4%) received baricitinib, and one patient each received tofacitinib or baricitinib (1/7, 14.3% each). Mean treatment duration was 33.4 months. GC were used in five patients (5/7, 71.4%), one patient received concomitant cyclosporine therapy and one patient received a combination of mepacrine and hydroxychloroquine. A summary of baseline characteristics, treatment and response is shown in Supplementary Table 1. A complete response was achieved by only one patient (1/7, 14.3%) under combination of baricitinib and cyclosporine A (5 mg/kg/d). Four patients (4/7, 57.1%) had a partial response and two (2/7, 28.6%) showed no response to therapy. For three patients (3/5, 60%) GC dose reduction was possible and one (1/5, 20%) successfully tapered GC.

Data on AEs was available for five patients (5/7, 71.4%), as follows: UTI (2/5, 40%), BK viruria (3/5, 60%), BK viremia (1/5, 20%). In neither case were dose reduction or therapy discontinuation reported. One case of herpes zoster (1/5, 20%) required intermittent JAKi dose reduction. Of all AEs, hospitalization was required in two cases (2/9, 22.2%): in one patient after multiple infectious events (clostridium difficile infection, pyelonephritis, urosepsis) and in one case of osteonecrosis. The latter discontinued JAKi therapy after 5.1 months due to this AE. Details on AEs are summarized in Table 2.

### Evidence on Effectiveness and Safety for Adult-Onset Still's Disease (AOSD)

For AOSD three case reports (54–56) and three case series (57–59) were identified (conference abstracts *n* = 2, letters to the editor *n* = 2, publications in full text *n* = 2).

A total of 26 patients were treated with a JAKi. Median age of JAKi initiation was 33 years (18–82 years). Most patients (18/26, 69.2%) were treated with tofacitinib, one patient (1/26, 3.8%) received ruxolitinib, and the other patients (7/26, 26.9%) baricitinib. Mean treatment duration was 7.6 months (1–24 months). Most patients (24/26, 92.3%) received GC either alone (7/26, 26.9%) or in combination with other disease modifying antirheumatic drugs (DMARDs) (17/26, 65.4%). Two patients had methotrexate (MTX) alone as supportive treatment (2/26, 7.7%). Mean GC dose at JAKi initiation was 37.3 mg prednisone equivalent per day. A summary of baseline characteristics, treatment and response is shown in Supplementary Table 1. A complete response was seen in eleven patients (11/26, 42.3%), with the same number of patients showing a partial response (11/26, 42.3%). No response was seen in a minority of patients (4/26, 15.4%). GC dosage at the end of follow-up was reported for twenty-two patients (22/24, 91.7%). Mean GC dose was 13.3 mg prednisone equivalent per day. GC dose reduction was possible for most patients (18/22, 81.8% of reported cases) and complete GC tapering was achieved by three patients (3/21, 14.3% of reported cases).

Data on AEs was available for 24 patients (24/26, 92.3%) and were overall rare: pneumonia (3/24, 12.5%) and menometrorrhagia (1/24, 4.2%); the latter required therapy discontinuation in one patient. One of the patients with bacterial pneumonia died after a 217-day long hospital stay. Otherwise no AEs required hospitalization. Details on AEs are summarized in Table 2.

### Evidence on Effectiveness and Safety for Systemic Juvenile Idiopathic Arthritis (SJIA)

Two case reports (60, 61) and one case series (57) (published as conference abstracts *n* = 1, letters *n* = 1, in full text *n* = 1) were identified.

Four patients with sJIA were treated with a JAKi. Median age at JAKi initiation was 9 years (4–13 years). Two patients received ruxolitinib (2/4, 50%), and one each received tofacitinib or baricitinib (1/4, 25% each). Mean treatment duration was 14.2 months (8–25 months). All received GC as supportive treatment. Two patients (2/4, 50%) received GC only along JAKi, and two patients—in combination with non-steroidal anti-inflammatory drugs and two patients (2/4, 50%). Data on baseline characteristics, treatment and response are shown in Supplementary Table 1. **Two** patients showed a complete response to therapy (2/4, 50%) and for the other two (2/4, 50%) a partial response was reported. GC dose reduction was possible for three patients (3/4, 75%) and one (1/4, 25%) successfully tapered GC to discontinuation.

Data on AEs was available for three patients (3/4, 75%), and none were reported (Table 2).

### Evidence on Effectiveness and Safety for FMF

Two case series (62, 63) and one case report (64) (full text *n* = 2, letters *n* = 1) were identified.

A total of six patients with FMF were treated with a JAKi. Median age at JAKi initiation was 35.5 years (16–64 years). All patients were treated with tofacitinib 10 mg/d. Mean treatment duration was 4.5 months (2–12 months). One patient received no supportive treatment (1/6, 16.7%), one (1/6, 16.7%) received a combination of GC, colchicine, and sulfasalazine. Four patients (4/6, 66.7%) were prescribed colchicine. A summary of baseline characteristics, treatment and response is shown in Supplementary Table 1. A complete response was shown by half of the patients (3/6, 50%). The other three patients (3/6, 50%) developed no further flares, but acute phase reactants remained elevated, thus only a partial response was achieved.

Data on AEs was available for four patients (4/6, 66.7%) with none reported (Table 2).

### Evidence on Effectiveness and Safety for Behçet's Syndrome

Only one publication on BS could be identified (65). Thirteen patients were treated with a JAKi. Median age at JAKi initiation was 42 years (22–73 years). All patients received tofacitinib 10 mg/d. Patients were treated for a mean duration of 10.8 months (5–21 months). All but one patient received concomitant GC therapy (12/13, 92.3%). Tofacitinib was administered as additional therapy to other drugs such as azathioprine, thalidomide, leflunomide, colchicine, salazosulfapyridin. A summary of baseline characteristics, treatment and response is shown in Supplementary Table 1.

According to the criteria used in this systematic review, most patients achieved a partial response (8/13, 61.5%). Five patients showed no response to therapy (5/13, 38.5%), of whom one patient's condition worsened (1/13, 7.7%) and tofacitinib was withdrawn after 9 months of treatment. Two cases of herpes zoster reactivation were observed, both of which led to discontinuation of tofacitinib. No other AEs were reported (Table 2).

### Evidence on Effectiveness and Safety for Other Syndromes

No articles regarding CAPS, TRAPS, PFAPA, PAPA or MKD could be identified.

## Discussion

To our knowledge, this is the first systematic review analyzing the safety and effectiveness of JAKi in AID. The overview of the available clinical evidence is based on observational studies such as case reports and case series. Effectiveness was evaluated based on clinical response, defined by the authors of this systematic review as complete, partial or no response depending on (complete) symptom resolution and/or normalization of laboratory parameters for inflammation. Furthermore, AEs were described.

### Type I Interferonopathies

Most reports (*n* = 25) included in this systematic review investigated the use of JAKi for type I interferonopathies. Interferonopathies represent a group of rare monogenic AID, characterized by a disturbed control of interferon-mediated immune responses, especially of type I interferons. JAKi are potent inhibitors of the JAK-STAT pathway, involved in interferon signaling (66). Thus, the application of JAKi in interferonopathies seems rational. In this analysis, reports on SAVI, CANDLE, AGS and other interferonopathies were included.

Fifty-two patients (CANDLE *n* = 14, SAVI *n* = 28, AGS *n* = 3, other interferonopathies *n* = 7) could be identified. Overall promising results were seen: seven patients (7/52, 13.5%) showed a complete response to therapy, the majority (35/52, 67.3%) showed a partial and a minority (10/52, 19.2%) showed no treatment response.

For AGS, one publication (50) was identified but individual patient data was not available. This article reported preliminary results of an open-label single center study involving 35 patients with molecularly confirmed AGS. This is to our knowledge the largest AGS cohort treated with JAKi. All patients received baricitinib. The authors report overall improvement of daily diary scores within 1 month of therapy initiation. Neurological function was evaluated based on key developmental milestones, with 20 patients (20/35, 57.1%) meeting new milestones and 12 (12/35, 34.3%) gaining two to seven new skills. In the three cases presented in this systematic review, neurological symptoms were leading in just one case (49) showing improvement under ruxolitinib. Regarding the safety profile of JAKi, in this AGS cohort (50) only one case of BK viremia was described—a relatively frequent event in interferonopathy patients discussed here (10/52, 19.2%).

Notably, amongst interferonopathy patients, JAKi was most efficient for CANDLE patients: complete remission was achieved by six patients (6/14, 42.6%). Additionally, a GC sparing effect in this group is suggested by the available data, since seven patients (7/11, 63.6%) were able to discontinue GC and in four patients (4/11, 36.4%) GC dose reduction was possible.

One hypothesis for the difference in treatment outcome between interferonopathies is that the better treatment response is owed to a higher JAKi dosage. A direct comparison is difficult since mostly pediatric patients were treated and JAKi dosage was reported as mg per kilogram without documenting weight for each individual patient. Mean baricitinib and tofacitinib dosages in CANDLE cases were 6.8 and 5 mg/d, respectively. Mean baricitinib doses for SAVI were 6 mg/d (reported for 6/7, 85.7%) up to a mean 8 mg/d in other interferonopathies. Most SAVI patients were treated with ruxolitinib at a mean dose 10.8 mg/d (reported for 13/18, 72.2% patients treated).

A head-to-head comparison of effectiveness is difficult due to (1) the small number of patients treated (2) the different choice of JAKi and the respective dosage used and (3) partial missing individual data.

Regarding safety it should be mentioned that most infectious AEs in this analysis occurred in patients with type I interferonopathies: seven cases of pneumonia (7/10, 70%), all UTIs (17/17, 100%) and all cases of BK viremia and viruria (10/10 and 4/4, respectively; 100%) (Table 2). The proportion of patients in this group who experienced any AE is also greater compared to other groups: around 17% of AOSD patients (4/24, 16.7%), even less in BS patients (2/13, 15.4%) and none of the FMF and sJIA patients.

Of overall 59 AEs reported, 52 (88.1%) were due to infections (Table 2). In general JAKi show a heterogenous risk of infectious complications. For example, a known class effect for JAKi is an elevated risk of herpes zoster (67–70). In one recent study, serious infections were more frequent with tofacitinib at a dose of 10 mg twice daily compared to TNF inhibition (71), which contradicts some available evidence pointing to a similar risk of serious infections under JAKi compared to other biological disease-modifying antirheumatic drugs (bDMARDs) (72, 73). The risk for opportunistic infections (herpes zoster, tuberculosis) under tofacitinib in this study was higher compared to a TNF-inhibitor, and even more so when tofacitinib dose was 10 mg compared to 5 mg. In this systematic review two cases of herpes infections occurred under tofacitinib and baricitinib each. One case occurred under ruxolitinib.

A controversially discussed adverse effect of JAKi are thromboembolic events. This risk has been shown to be elevated relative to TNF inhibitors, along with a clinically meaningful risk of serious heart-related AEs, cancers, blood clots and death in older patients with RA (71). However, several (meta-) analyses did not provide evidence supporting an increased risk of thromboembolism with JAKi (67, 74, 75). In this systematic review, thromboembolic events were not reported.

The discrepancy in the frequency of AEs in the different disease groups here could reflect the inconsistency in reporting of AEs, commented further below. It should be considered that for FMF, sJIA and Behçet's syndrome only a few reports were available for analysis. However, one reason for the higher incidence of infections amongst interferonopathy patients might be due to a dose dependent effect. All FMF and Behçet's syndrome patients, and most AOSD patients received a “standard dose” of tofacitinib (5 mg/d) or baricitinib (4 mg/d). As mentioned above, JAKi doses varied amongst interferonopathy patients. Nevertheless, baricitinib was often administered at doses higher than 4 mg/d—up to a mean 6.8 mg/d in CANDLE patients and a mean 6 mg/d for SAVI patients. Notably, most interferonopathy patients were pediatric patients, suggesting a higher dose pro kilogram body weight.

Another hypothesis is that interferonopathy patients generally have a higher risk for infections. While infections can be considered potential triggers for disease onset or flares (5), a predisposition for infections in interferonopathy patients is currently not proven. However, for other diseases with a prominent interferon signature, such as systemic lupus erythematosus (SLE) and dermatomyositis, a susceptibility for infections has been reported (76, 77). Thus, a possible explanation for the elevated incidence of infectious AEs in this subgroup could be an intrinsically dysfunctional immune system leaving patients exposed to an increased risk of infection.

### Other AID

Part of the systematic database searches about JAKi for treating monogenic AID were CAPS, TRAPS MKD and FMF. Of those, publications were identified only for FMF. In FMF patients, JAKi resulted in a complete response in half of the patients and a partial response for the rest (3/6, 50% each). Eleven AOSD patients had a complete response and the same number of patients a partial response (11/26, 42.3% each). Two sJIA patients completely responded to JAKi therapy (2/4, 50%) and for the other two (2/4, 50%) a partial response was reported. Amongst BS a partial response was achieved by most (8/13, 61.5%), and five (5/13, 38.5%) showed no response to therapy.

Although JAKi did not lead to complete remission in all AOSD patients, the majority of them were able to taper or withdraw GC—used as supportive treatment in most patients (24/26, 92.3%). Dosage of GC at last follow up was reported for 22 patients. Mean GC dose at JAKi initiation was 37.3 mg prednisone equivalent per day, and 13.3 mg/d at last follow-up. This highlights the potential of JAKi as GC sparing drug in AOSD, especially in patients with articular phenotype. The majority of AOSD patients presented with arthritis (20/26, 76.9%), of whom most showed a complete or a partial response (8/20, 40% for each group). Additionally, all FMF patients included in this analysis had active arthritis. All of them showed clinical improvement under JAKi. Therefore, it can be hypothesized that JAKis are especially beneficial for patients with active arthritis.

### Limitations and Considerations for the Future

Due to the rare nature of AID and the relatively recent availability of JAKi, there are currently no RCTs available, and most publications included were case reports or series. Therefore, conducting a risk of bias assessment was not possible. Many authors were contacted to complete missing data, however in some instances despite our best effort complete information could not be obtained. In the analyzed publications a relatively low systematization in conduct and reporting was observed, which in part resulted in limited details on individual patient characteristics at baseline and post JAKi treatment. In order to include as much information as possible on the topic, congress abstracts were also included in the analysis. Abstracts are generally considered to potentially lower the overall evidence level in a systematic review. Therefore, only abstracts providing sufficient clinical data were included in the final analysis (78).

To present the results of this systematic review, a classification based on clinical symptoms and laboratory parameters was performed. Accordingly, treatment response was classified as complete, partial or none. Although this approach has not been validated, it has been previously used by other investigators (57–59) and serves as base for objectifying and summarizing the available evidence. The body of evidence found did not suffice for quantitative analysis due to its heterogeneity. Instead, an extensive narrative synthesis was conducted.

To this date, no universal criteria for reporting outcomes in AID patients exist. To improve and standardize reporting on treatment strategies in AID we suggest the following type of reporting (Table 3). clinical symptoms, inflammatory parameters, concomitant diseases, previous therapies; for the use of JAKi—exact dose, as well as information on any supportive treatment, including dosage; for a precise evaluation therapeutic response statements on dynamics of clinical symptoms, as well as inflammatory parameters should be noted. AEs especially infections should be closely monitored. In the publications included in this systematic review reports on AEs were sometimes insufficient—those were not documented in around 15% of cases (83/101, 82.2%).

**Table 3:** Suggestions for future reporting on treatment outcome.

**Pre-JAKi**	**JAKi**	**Post-JAKi**
**Clinical**		**Clinical**
Clinical symptoms	Dosage	Change in clinical symptoms
Disease score (if available)	Supportive treatment (including dosage)	Change in disease score (if available)
Concomitant diseases	Treatment duration	Change in dosage of supportive drugs (e.g. GC)
Previous therapies		Adverse events
**Inflammatory markers**		**Inflammatory markers**
CRP		CRP
ESR		ESR
others (complete blood count, ferritin, IFN gene expression, if applicable)		others (complete blood count, ferritin, IFN gene expression, if applicable)

Furthermore, disease (specific) activity scores and response criteria to compare AID studies are urgently needed. For monogenetic inflammasomopathies (FMF, CAPS, TRAPS, MKD) the Auto-Inflammatory Diseases Activity Index (AIDAI) (79) is a validated score but was only reported in one study concerning JAKi use in FMF (63). An EULAR task force is currently preparing specific criteria for AOSD which should be applied for future reporting (80). Regarding type I interferonopathies Frémond, M. et al., 2016 suggested a disease activity score for SAVI patients: the Disease Activity Rating Scale of TMEM173-mutated patients (42). This score for SAVI needs to be validated and scores for the other interferonopathies need to be developed. Overall, given their rare nature, a considerable number of AID patients treated with a JAKi (101) could be identified. The available evidence showed most patients did respond to JAKi therapy. This review was conducted to summarize the available evidence on new therapeutic possibilities for AID patients and to highlight the need for well-designed clinical trials investigating JAKi in AID. Currently, one phase 3 clinical trial investigating baricitinib in CANDLE, SAVI and AGS is being conducted (81). Research is actively underway in the direction of sJIA with two ongoing phase 3 randomized double-blind, placebo-controlled studies on baricitinib and tofacitinib (82, 83).

## Conclusion

This systematic review provides results from observational studies showing first pieces of evidence on treatment effectiveness of JAKi for AID. To validate these results and confirm efficacy and safety of JAKi for specific AID, clinical trials need to be initiated.

## Data Availability Statement

The original contributions presented in the study are included in the article/Supplementary Material, further inquiries can be directed to the corresponding author/s.

## Author Contributions

ZB, GB, and MK: systematic review concept and design. ZB, NR, and MK: systematic review protocol. ZB: database search. ZB and AP: study selection. MK: arbiter during publications screening. ZB: data extraction and synthesis. ZB and MK: manuscript drafting. ZB, MK, NR, GB, and AP: manuscript revision and final review. All authors approved the final version.

## Conflict of Interest

MK has received speakers and consultant honoraria by Abbvie, Pfizer, Galapagos and Lilly. GB has received honoraria for lectures and consulting from Novartis, Sobi, Sanofi, as well as institutional grants from Novartis. The remaining authors declare that the research was conducted in the absence of any commercial or financial relationships that could be construed as a potential conflict of interest.

## Publisher's Note

All claims expressed in this article are solely those of the authors and do not necessarily represent those of their affiliated organizations, or those of the publisher, the editors and the reviewers. Any product that may be evaluated in this article, or claim that may be made by its manufacturer, is not guaranteed or endorsed by the publisher.
