# Supplementary material for: Effectiveness and Safety of JAK Inhibitors in Autoinflammatory Diseases: A Systematic Review
Source: Front Med (Lausanne). 2022 Jun 27;9:930071. doi: 10.3389/fmed.2022.930071 (PMC9271622; doi:10.3389/fmed.2022.930071)
Supplement: Supplementary file 1 [file Table_1.docx]

**Table 1** **Baseline characteristics, treatment, and treatment response in AID patients**

| Patient | Age at initiation, sex | Clinical symptoms | Drug | Dosage | Supportive Treatment | Duration (months) | CRP pre  (mg/l) | CRP post (mg/l) | Response |
| --- | --- | --- | --- | --- | --- | --- | --- | --- | --- |
| CANDLE | | | | | | | | | |
| 1 (29) | 5, m | dyspnea fever, rash, periorbital swelling, hypertrichosis, lipodystrophy, polyarthritis, atrophic facial musculature, cough, hepatomegaly, arterial hypertension | BAR | 6mg/d for 3 days, 8mg/d | GC | 18 | 43.04 | 2.8^4^ | Partial  no clinical symptoms, elevated CRP during follow-up |
| 2 (30) | 12, f | recurring purpuric annular plaques, recurrent fever, abdominal pain, myositis, panniculitis | TOF | 10mg/d 6 Mo; 20mg/d | GC | >48 | n/a | n/a | Partial  resolution of clinical symptoms, no laboratory parameters |
| 3 (32) | 17, f | skin rashes, arthritis, panniculitis, lipodystrophy, alopecia, growth delay | TOF | 10 mg/d | GC | 34 | 1.7 | 0.2^5^ | Complete |
| 4 (34) | n/a | n/a | BAR | 0.1mg/d titrated to 6mg/d | GC 0.84mg/kg/d | 149.1 | n/a | n/a | Partial  partial resolution of clinical symptoms*  GC reduction to 0.27  mg/kg/d |
| 5 (34) | n/a | n/a | BAR | 0.2mg/d titrated to 6mg/d | GC | 140.5 | n/a | <5 | Complete |
| 6 (34) | n/a | n/a | BAR | 1mg/d titrated to 6mg/d | GC | 127.5 | n/a | n/a | Partial  partial resolution of clinical symptoms*  GC reduction** |
| 7 (34) | n/a | n/a | BAR | 1mg/d titrated to 8mg/d | GC | 128.2 | n/a | <5 | Complete |
| 8 (34) | n/a | n/a | BAR | 1mg/d titrated to 9mg/d | GC | 123.9 | n/a | <5 | Complete |
| 9 (34) | n/a | n/a | BAR | 1mg/d titrated to 4mg/d | GC | 98.8 | n/a | n/a | Partial  partial resolution of clinical symptoms*  GC reduction** |
| 10 (34) | n/a | n/a | BAR | 1mg/d titrated to 4mg/d | GC | 67.5 | n/a | n/a | No |
| 11 (34) | n/a | n/a | BAR | 1mg/d titrated to 6mg/d | GC | 101.4 | n/a | n/a | Partial  partial resolution of clinical symptoms*  GC reduction** |
| 12 (34) | n/a | n/a | BAR | 3mg/d titrated to 10mg/d | GC | 89.5 | n/a | <5 | Complete |
| 13 (34) | n/a | n/a | BAR | 7mg/ titrated to 9mg/d | none | 75.2 | n/a | <5 | Complete |
| 14 (31) | 1y6mo, m | macular rash, fever, arthralgia, arthritis, growth delay, regression of milestones, myositis | TOF | 5mg/d | n/a | n/a | 142 | normal | Partial  partial resolution of clinical symptoms, normalization of laboratory parameters |
| SAVI | | | | | | | | | |
| 1 (41) | 13, m | fever, rash, lipodystrophy, polyarthritis, pulmonary hypertension | RUX | 10mg/d | n/a | 6 | 14.7 | 5.9 | Partial  only lung function and skin improvement |
| 2 (41) | 7, f | recurring purpuric annular plaques, fever, abdominal pain, myositis, panniculitis, pulmonary hypertension | RUX | 10mg/d | n/a | 6 | 7.6 | 3.19 | Partial  no information on clinical symptoms except “improvement” |
| 3 (44) | 37, m | erythema, livedo reticularis, alopecia, vasculitis, vasculitis ulcer | BAR | 4mg/d for 2 months; 6mg/d | Pred 0,2mg/kg | ongoing, >43 | n/a | n/a | Partial  ulcer - healed; improved well-being; persisting livedo, no laboratory improvement |
| 4 (46) | 10, f | fever, skin lesions, acral ulcers, arthralgia, ILD | RUX | 10mg/d; 15mg/d; 20mg/d | Pred 0,4mg/kg/d | >36 | 36 | 0 | Partial  GC withdrawal, resolution of skin lesions, lung function improvement |
| 5 (46) | 8, f | growth delay, livedo reticularis, ILD + oxygen support + NIV | RUX | 5mg/d; 10mg/d, 15mg/d, 5mg/d | Pred 0,3mg/kg/d | 25 | 4.8 | 4.9 | Partial  GC tapered to 0,2mg/kg/d; skin improvement; initial improvement in lung function, deterioration after 18 months |
| 6 (46) | 2, f | erythematosus vesicular rash, dermatitis, fever, cough, lungs - septal thickening on CT | RUX | 5mg/d, 15mg/d | None | 22 | 26.6 | 8.9 | Partial  resolution skin lesions, initial improvement in lung function and radiological findings, followed GC-responsive lung disease relapse |
| 7 (36) | 1, m | fever, livedoid skin lesions, ulcerated lesions on heels, ILD, oxygen dependent | BAR | 2mg/d | Mpred 1mg/kg/d | n/a | 79 | 1.5 | Partial  no oxygen dependency, resolution of skin lesions; GC tapered to 0,5mg/kg/d; no information on ILD; ESR elevated |
| 8 (40) | 2y9mo, m | pulmonary hypertension, hypoxia, livedo racemosa, vomiting, epistaxis, failure to thrive, growth delay | RUX | 5mg/d | Pred 2mg/kg/d | >12 | n/a | n/a | Partial  normalization pulmonary pressure; neurodevelopmental improvement; persistence of livedo reticularis |
| 9 (47) | 37, m | dyspnea, ILD, pneumonia, finger clubbing | RUX | 10mg/d | None | 4 | 55.3 | n/a | No  patient died (ILD, heart failure) |
| 10 (47) | 13, m | growth delay, finger clubbing, recurrent migratory polyarthritis | RUX | 5mg/d | None | 12 | n/a | n/a | No |
| 11 (45) | 1y1mo, m | dyspnea, cyanosis, finger clubbing, telangiectasis, Chilblain lesions, myositis, fever, growth delay | TOF | 5mg/d | Pred | 7 | n/a | n/a | No  patient died due to acute respiratory failure |
| 12 (45) | 5, m | cough, tachypnea, finger clubbing, arthritis | TOF | 5m/d | Pred | 20 | elevated | elevated | No |
| 13 (42, 43) | 4, f | fever, scalp lesions, fatigue, ILD | RUX | 5mg/d  10mg/d  5mg/d | Pred 0,5mg/kg/d | 18 | 50-100 | 10-20 | Partial  GC withdrawal, disease score improvement^1^, occasional fever episodes |
| 14(42) (43) | 8, m | ulcers, nail dystrophy, fatigue, ILD | RUX | 5mg/d; 10mg/d; 15mg/d; 20mg/d | Pred 0,6mg/kg/d | 16 | 6 | 6 | Partial  GC withdrawal, general improvement, disease score improvement^2^, persisting ulcer |
| 15 (42, 43) | 12, m | fever, fatigue, ILD, erythematous skin lesions | RUX | 10mg/d | Pred 0,2mg/kg/d  HCQ 11mg/kg/d | 6 | 49 | 19 | Partial  GC withdrawal; skin lesion improvement, weight gain,  disease score improvement^3^ |
| 16 (37) | 5y10mo, f | nasal septum perforation, growth delay; ILD; | BAR | n/a | n/a | 9 | n/a | n/a | No |
| 17 (35) | 8 mo, m | recurrent skin lesions, fever, ischemic changes of digits | RUX | 2mg/d; 5mg/d | n/a | >6 | n/a | n/a | Partial  less skin lesions, less hospital admissions |
| 18 (39) | 6, f | fever, cough, dyspnea, Raynaud, arthralgia, rash | TOF | 7.5mg/d | Pred 0.7mg/kg/d | 12 | n/a | normal | Partial  GC withdrawal |
| 19 (38) | 18, m | fever, dyspnea, ILD, chilblain lupus-like lesions, rash, livedo reticularis | RUX | 5mg/d; 20mg/d | n/a | 2.5 | n/a | n/a | No  patient died (ILD) |
| 20 (34) | n/a | n/a | BAR | 2mg/d titrated to 6mg/d | Pred | 80.9 | n/a | n/a | No |
| 21 (34) | n/a | n/a | BAR | 7mg/d titrated to 10mg/d | None | 74.4 | n/a | n/a | Partial  partial resolution of clinical symptoms* |
| 22 (34) | n/a | n/a | BAR | 3mg/d titrated to 6mg/d | None | 74.5 | n/a | n/a | Partial  partial resolution of clinical symptoms* |
| 23 (34) | n/a | n/a | BAR | 3mg/d titrated to 6mg/d | None | 46.3 | n/a | n/a | Partial  partial resolution of clinical symptoms* |
| 24 (42) | 14, m | dyspnea, ILD, rash, end-stage pulmonary failure; | RUX | 0.28mg/kg/d | GC 0.16mg/kg/d | 2.5 | n/a | n/a | partial  weight and height increase, significant disease score reduction***  patient died after humoral rejection after lung transplant for severe ILD |
| 25 (42) | 7, m | extreme vasculopathy, dyspnea at rest, ILD, arthritis, myositis | RUX | 1.10mg/kg/d | GC 0.625mg/kg/d  IVIG | 41 | n/a | n/a | partial  weight and height increase, significant disease score reduction*** |
| 26 (42) | 12, f | severe dyspnea at rest, oxygen therapy, ILD, severe polyarthritis | RUX | 0,2mg/kg/d | GC 1.5mg/kg/d IVIG | 17 | n/a | n/a | partial  weight and height increase, significant disease score reduction*** |
| 27 (42) | 8, f | fever, severe vasculopathy, dyspnea on moderate exercise; ILD, severe polyarthritis | RUX | 0,83mg/kg/d | GC 0.22mg/kg/d, ETA, monthly GC pulses | 18 | n/a | n/a | partial  weight and height increase, significant disease score reduction*** |
| 28 (42) | 7mo | fever, severe dyspnea at rest, ILD | RUX | 1mg/kg/d | IVIG | 24 | n/a | n/a | partial  weight and height increase, significant disease score reduction*** |
| AGS | | | | | | | | | |
| 1 (48) | 22, f | severe chilblains | BAR | 2mg/d | n/a | >18 | n/a | n/a | Partial  resolution of chilblains, no laboratory parameters |
| 2 (50) | 11, m | rash,  dyspnea, ILD, lower limb weakness, calcifications in cerebral cortex and basal ganglia | TOF | 10mg/d | CsA + GC | 24 | 3.1 | 7.8 | Partial  rash resolution, improvement of pulmonary function; persisting muscle weakness; GC dose reduction |
| 3 (49) | 1year 6 months, m | neurological (irritability, sleep disturbances, language regression, loss of postural control, axial hypotonia, extrapyramidal signs, microcephaly) | RUX | 0,8mg/kg/d | Monthly IVIG 1mg/kg | ongoing, >43 | normal | normal | Partial  progressive clinical improvement***** |
| Other type I interferonopathies | | | | | | | | | |
| 1 (34) | n/a | n/a | BAR | 0.5mg/d titrated to 8mg/d | GC | 20.3 | n/a | n/a | No |
| 2 (34) | n/a | n/a | BAR | 1mg/d titrated to 6mg/d | GC | 93.9 | n/a | n/a | Partial  partial resolution of clinical symptoms*  GC reduction** |
| 3 (34) | n/a | n/a | BAR | 3mg/d titrated to 9mg/d | GC | 5.1 | n/a | n/a | No |
| 4 (34) | n/a | homozygous SAMHD1 deletion  n/a | BAR | 3mg/d titrated to 9mg/d | none | 68.7 | n/a | n/a | Partial  partial resolution of clinical symptoms*  GC reduction** |
| 5 (52) | 1 mo, m | novel type I interferonopathy: de novo PSMB9 p.G156D mutation  rash, fever, respiratory failure, pulmonary hypertension, cluster seizures | TOF | 0.2mg/kg/d 2 weeks, 0,3mg/kg/d | none | 6 | n/a | <5 | Partial  rash improvement, ECMO withdrawal, pulmonary hypertension "controlled" (SIL, MAC) |
| 6 (53) | 17, m | DNase II deficiency  weakness, palpitations, dyspnea, joint stiffness, headache | RUX | 15mg/d; 20mg/d; 15mg/d | MEP 100mg + HCQ 200mg + GC | 29 | >20 | n/a | Partial  resolution of pulmonary hypertension, no laboratory parameters |
| 7 (54) | 4, m | novel type I interferonopathy: PSMB8 mutation  fever, rash, abdominal pain, hepatosplenomegaly, cervical lymphadenopathy | BAR | 8mg/d | CsA + GC | 11 | elevated | normal | Complete |
| AOSD | | | | | | | | | |
| 1 (55) | 18, m | fever, rash sore throat, synovitis | TOF | 10mg/d | MTX 20mg/week | 6 | elevated | n/a | Partial  no laboratory parameters |
| 2 (55) | 31, m | fever, rash sore throat, synovitis | TOF | 10mg/d | MTX 20mg/week | 6 | elevated | n/a | Partial  no laboratory parameters |
| 3 (59) | 33, f | polyarthritis, rash | TOF | 10mg/d | Pred 40mg/d | 24 | 70-80 | 20-30 | Complete |
| 4 (59) | 27, f | Fever, polyarthritis | TOF | 10mg/d | Pred 60mg/d + MTX | 13 | 20 | 20 | Complete |
| 5 (59) | 32, f | fever, rash, pharyngitis, myalgia | TOF | 10mg/d | Pred 50mg/d + HCQ | 12 | 70-80 | <10 | Complete |
| 6 (59) | 58, f | polyarthritis, rash | TOF | 10mg/d | Pred 15mg/d + MTX + HCQ | 6 | <10 | <10 | Complete |
| 7 (59) | 35, f | polyarthritis, rash | TOF | 10mg/d | Pred 15mg/d + MTX | 1 | <20 | <10 | Partial  persistence of clinical symptoms |
| 8 (59) | 29, f | polyarthritis, early joint destruction, lymphadenopathy, MAS | TOF | 10mg/d | Pred 60mg/d + MTX | 9 | >80 | <10 | Complete |
| 9 (59) | 82, f | ESR elevation | TOF | 5mg/d | Pred 25mg/d + HCQ | 9 | 40 | <10 | Complete |
| 10 (59) | 25, f | polyarthritis | TOF | 10mg/d | Pred 50mg/d + MTX | 4 | 20-30 | <10 | Partial  persistence of clinical symptoms |
| 11 (59) | 41, f | polyarthritis | TOF | 10mg/d | Pred 60mg/d + MTX | 5 | <10 | <10 | Partial  persistence of clinical symptoms |
| 12 (59) | 31, f | polyarthritis | TOF | 5mg/d | Pred 20mg/d + MTX + HCQ + CsA | 4 | 10-20 | <10 | Complete |
| 13 (59) | 33, f | fever, rash, pharyngitis, myalgia, polyarthritis | TOF | 10mg/d | Pred 40mg/d + MTX + HCQ | 3 | 70-80 | 60 | Complete |
| 14 (59) | 35, m | MAS | TOF | 10mg/d | Pred 22.5mg/d + CsA + ANA | 1 | 60 | 10 | Partial  persistence of clinical symptoms |
| 15 (59) | 18, m | polyarthritis, rash | TOF | 10mg/d | Pred 15mg/d + HCQ | 1 | 20 | 50 | Partial  clinical improvement, CRP elevated |
| 16 (59) | 18, f | polyarthritis, rash, MAS | TOF | 10mg/d | Pred 50mg/d + CsA + MTX | 1 | 5 | 10 | Partial  persistence of clinical symptoms |
| 17 (56) | 68, f | fever, arthritis | TOF | 5mg/d | Pred 30mg/d | 6 | 14.8 | 7.3 | Partial  "clinical and serological improvement", occasional CRP elevations and hyperferritinemia during follow-up; patient died (bacterial pneumonia) |
| 18 (60) | 43, f | fever, arthritis, synovitis, rash, pharyngitis, serositis, splenomegaly | BAR | 4mg/d | Pred 10mg/d | 15 | 231 | <5 | Complete |
| 19 (60) | 32, m | fever, polyarthritis, rash, synovitis | BAR | 4mg/d | Pred 40mgd, MTX 20mg/week | 9 | 94 | 56 | No |
| 20 (60) | 63, f | persistent fever | BAR | 4mg/d | Pred 15mg/d | 9 | 48 | 2 | Complete |
| 21 (57) | 50, f | polyarthritis | BAR | 4mg/d | Mpred 9mg/d + ANA | 9 | 3.8 | <5 | Complete |
| 22 (58) | 28, m | fever, polyarthritis, rash | BAR | 4mg/d | Pred 80 mg/d | 1 | 60 | <25 | No |
| 23 (58) | 32, m | fever, polyarthritis, rash | BAR; UPA | 4mg/d; 15mg/d | Pred 16mg/d; MTX 20mg/week (stop), ANA 100mg/d; COL 1.5 mg/d | 22 | 230-250 | 0 | Partial  incomplete resolution of clinical symptoms |
| 24 (58) | 40, f | fever, polyarthritis, rash | RUX | 30mg/d | Pred 60mg/d; ANA 200mg/d | 9 | 50 | 0 | No |
| 25 (58) | 48, f | fever, polyarthritis, rash | TOF | 10mg/d | Pred 50mg/d | 12 | 20 | <25 | Partial  CRP elevated |
| 26 (58) | 50, f | fever, polyarthritis, rash | BAR | 4mg/d | Pred 60 mg/d | 1 | 150-200 | 50-70 | No |
| sJIA | | | | | | | | | |
| 1 (58) | 6, f | fever, polyarthritis, rash | RUX | 10mg/d; 30mg/d | Pred 3mg/d, IND | 25 | 30-50 | <10 | Partial  incomplete resolution of clinical symptoms |
| 2 (58) | 12, f | fever, polyarthritis | BAR | 4mg/d; 8 mg/d | Pred 40mg/d, NAP | 9 | 130-150 | <10 | Partial  incomplete resolution of clinical symptoms |
| 3 (61) | 4, f | recurrent fever, urticaria, arthralgia, ILD | RUX | 1mg/kg/d | Pred 0,5mg/kg/d; Mpred pulse 1x/Mo (3 times) | 15 | n/a | normal | Complete |
| 4 (62) | 13, f | polyarthritis, axillary lymphadenopathy | TOF | 5mg/d; 10mg/d | Mpred 4mg/d | 8 | 127 | 30 | Complete |
| FMF | | | | | | | | | |
| 1 (63) | 16, m | recurrent fever, generalized peritonitis, monoarthritis, rash, | TOF | 10mg/d | n/a | 2 | elevated | normal | Complete |
| 2 (65) | 27, f | fever, polyarthritis, peritonitis | TOF | 10mg/d | SSZ + GC + COL ^4^ | 12 | 29.1 | 31.4 | Partial  attack-free, CRP elevated |
| 3 (64) | 28, m | fever, peritonitis, serositis, arthritis, proteinuria, AA amyloidosis | TOF | 10mg/d | COL 3mg/d | 3 | 12.4 | 6 | Complete |
| 4 (64) | 58, f | peritonitis, fever, pleuritis, arthritis | TOF | 10mg/d | COL 1.5mg/d | 3 | 14 | 4 | Complete |
| 5 (64) | 64, f | fever, arthritis | TOF | 10mg/d | COL 1.5mg/d | 4 | 98 | 11 | Partial  attack-free, CRP elevated |
| 6 (64) | 43, f | fever, peritonitis, arthritis | TOF | 10mg/d | COL 2.5mg/d | 3 | 23 | 11.6 | Partial  attack-free, CRP elevated |
| BS | | | | | | | | | |
| 1 (66) | 37, m | oral ulcers, skin involvement, aortic aneurysm | TOF | 10mg/d | GC, AZA, CYC, LEF, COL, TCZ | 19 | n/a | n/a | Partial  resolution of ulcers, no newly  onset imaging/endoscopic findings |
| 2 (66) | 42, m | oral ulcers, genital ulcers, skin involvement, aortic aneurysm | TOF | 10mg/d | GC, THA, CsA, AZA, CYC | 5 | n/a | n/a | Partial  resolution of ulcers, no newly  onset imaging/endoscopic findings |
| 3 (66) | 29, f | oral ulcers, pulmonary embolism | TOF | 10mg/d | GC, MMF, CYC, LEF, COL | 4 | n/a | n/a | Partial  resolution of ulcers, no newly  onset imaging/endoscopic findings |
| 4 (66) | 42, f | oral ulcers, genital ulcers, skin involvement, aortic valve regurgitation | TOF | 10mg/d | GC, CYC | 19 | n/a | n/a | Partial  resolution of ulcers, no newly  onset imaging/endoscopic findings |
| 5 (66) | 64, m | oral ulcers, genital ulcers, aortic valve regurgitation, aortic aneurysm | TOF | 10mg/d | GC, LEF | 6 | n/a | n/a | Partial  resolution of ulcers, no newly  onset imaging/endoscopic findings |
| 6 (66) | 42, m | oral ulcers, genital ulcers, polyarthritis | TOF | 10mg/d | SSZ, LEF, THA | 21 | n/a | n/a | Partial  resolution of ulcers, no newly  onset imaging/endoscopic findings |
| 7 (66) | 30, m | oral ulcers, GI ulcers, skin involvement, scleritis, polyarthritis | TOF | 10mg/d | GC, SSZ, MTX, AZA, COL, THA | 7 | n/a | n/a | Partial  resolution of ulcers, no newly  onset imaging/endoscopic findings |
| 8 (66) | 73, m | oral ulcers, genital ulcers, GI ulcers | TOF | 10mg/d | GC; CYC, SSZ | 8 | n/a | n/a | Partial  resolution of ulcers, no newly  onset imaging/endoscopic findings |
| 9 (66) | 59, f | oral ulcers, GI ulcers | TOF | 10mg/d | GC, CYC, SSZ, THA | 9 | n/a | n/a | No |
| 10 (66) | 48, m | oral ulcers, genital, GI ulcers | TOF | 10mg/d | GC, TAC, SSZ | 7 | n/a | n/a | No |
| 11 (66) | 22, f | oral ulcers, genital ulcers, GI ulcers | TOF | 10mg/d | GC, SSZ, THA, MTX | 21 | n/a | n/a | No |
| 12 (66) | 37, f | oral ulcers, genital ulcers, GI ulcers, MDS | TOF | 10mg/d | GC, TAC, THA, COL | 5 | n/a | n/a | No |
| 13 (66) | 23, f | oral ulcers, genital ulcers, GI ulcers | TOF | 10mg/d | GC, CYC, AZA, THA | 9 | n/a | n/a | No  worsening of clinical symptoms |

*Clinical symptoms were summarized in a daily diary score (DDS). A score of <0.15 showed complete resolution, <0.5 (or <1 for SAVI) was considered as partial resolution of clinical symptoms (34)

** prednisone ≥50% dose reduction or 0.15mg/kg/d

*** p<0.05; Disease score parameters: fever; skin, nail, and hair lesions; respiratory difficulties and fatigue

**** non-significant

***** confirmed on standardized evaluations (Griffiths-III Developmental Scale, Gross Motor Function Classification System)

^1^Disease score at JAKi initiation 12 vs 2 (after 12 months)

^2^Disease score at JAKi initiation 10 vs 2.8 (after 12 months)

^3^Disease score at JAKi initiation 11.2 vs 5 (after 6 months)

^4^1 month after baricitinib initiation

^5^ time point unclear

*n/a* not available *ILD* idiopathic lung disease *NIV* non-invasive ventilation *MDS* myelodysplastic syndrome

***BAR*** baricitinib ***TOF*** tofacitinib ***RUX*** ruxolitinib ***UPA*** upadacitinib ***Pred*** prednisolone ***Mpred*** methylprednisolone ***HCQ*** hydroxychloroquine ***CsA*** cyclosporine A ***MEP*** mepacrine ***CYC*** cyclophosphamide ***IND*** indomethacin ***NAP*** naproxen ***TAC*** tacrolimus ***SSZ*** sulfasalazine ***THA*** thalidomide ***AZA*** azathioprine ***MTX*** methotrexate ***COL*** colchicine ***ETA*** etanercept ***LEF*** leflunomide ***TCZ*** tocilizumab ***ANA*** anakinra ***IVIG*** intravenous immunoglobulin ***SIL*** sildenafil ***MAC*** macitentan
